# Why Do Heterosporous Plants Have So Few Chromosomes?

**DOI:** 10.3389/fpls.2022.807302

**Published:** 2022-02-16

**Authors:** Sylvia P. Kinosian, Carol A. Rowe, Paul G. Wolf

**Affiliations:** ^1^Negaunee Institute for Plant Conservation Science, Chicago Botanic Garden, Glencoe, IL, United States; ^2^Earth System Science Center, The University of Alabama in Huntsville, Huntsville, AL, United States; ^3^Department of Biological Sciences, The University of Alabama in Huntsville, Huntsville, AL, United States

**Keywords:** ferns, homospory, genome evolution, meiosis, heterospory, chromosome evolution

## Abstract

The mechanisms controlling chromosome number, size, and shape, and the relationship of these traits to genome size, remain some of the least understood aspects of genome evolution. Across vascular plants, there is a striking disparity in chromosome number between homosporous and heterosporous lineages. Homosporous plants (comprising most ferns and some lycophytes) have high chromosome numbers compared to heterosporous lineages (some ferns and lycophytes and all seed plants). Many studies have investigated why homosporous plants have so many chromosomes. However, homospory is the ancestral condition from which heterospory has been derived several times. Following this phylogenetic perspective, a more appropriate question to ask is why heterosporous plants have so few chromosomes. Here, we review life history differences between heterosporous and homosporous plants, previous work on chromosome number and genome size in each lineage, known mechanisms of genome downsizing and chromosomal rearrangements, and conclude with future prospects for comparative research.

## Introduction

The nuclear genetic material of eukaryotes is contained within chromosomes. The number, length, and centromere location of chromosomes in an organism (the karyotype) varies considerably among lineages (Ohno, 1984; Schubert and Lysak, 2011). In plants, chromosome number is often phylogenetically conserved (Manton, 1950; Wagner and Wagner, 1979; Weiss-Schneeweiss and Schneeweiss, 2013). Changes in chromosome number or structure can alter the balanced chromosome pairing that is critical for cell division, leading to sexual sterility or death. Thus, an understanding of how plant chromosome numbers evolve has implications for plant reproductive biology, systematics, and genome evolution, among other processes (Haufler, 2002; Li Z. et al., 2020; Fujiwara et al., 2021).

**FIGURE 1:**
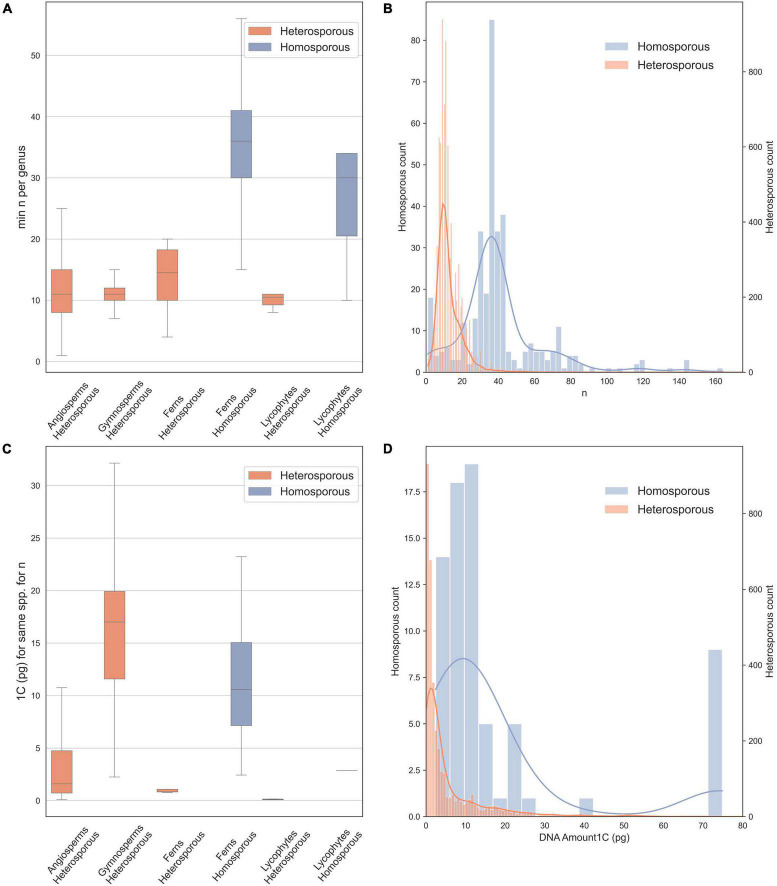
Chromosome counts and genome sizes for heterosporous and homosporous plant clades. Data were downloaded in January 2021 from the Chromosome Counts Database version 1.47 (Rice et al., 2015) and the Plant DNA *C*-values Database (Pellicer and Leitch, 2020). **(A)** Boxplots of chromosome counts (*n* = 7,900), including only the minimum reported count for a genus, boxes are central two quartiles and whiskers are interquartile range × 1.5 (data from Rice et al., 2015). **(B)** Histogram with kernel density plot of chromosome counts for homosporous plants (blue, right *y*-axis; mean *n* = 42.25) and heterosporous plants (orange, left *y*-axis; mean *n* = 12.74)—note scales are different to account for unequal sample size. **(C)** Boxplots of genome size estimates (1C), included are all possible minimum values for each species. **(D)** Histogram with kernel density plot of genome size estimates for homosporous plants (blue, right *y*-axis; mean 1C = 12.52) and heterosporous plants (orange, left *y*-axis; mean 1C = 6.43), on different scales.

**FIGURE 2:**
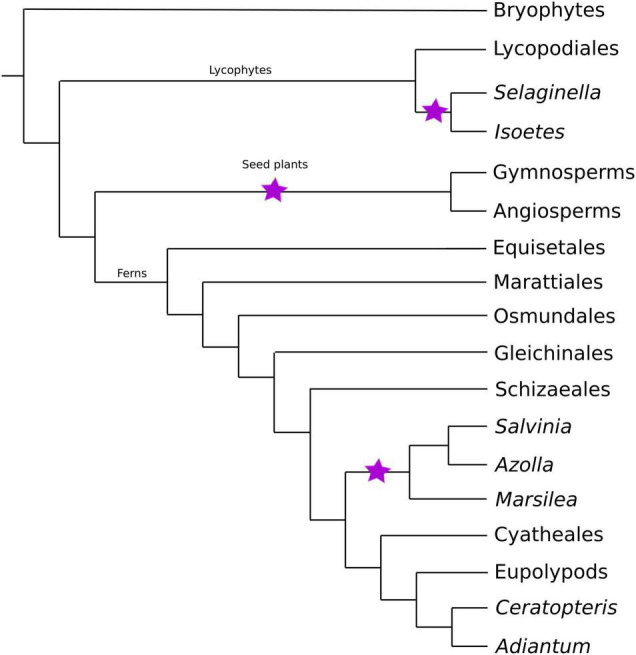
Land plant phylogeny (PPG, 2016). The three major clades of vascular plants (lycophytes, seed plants, and ferns) are shown, along with the three heterosporous lineages denoted with purple stars. Tip labels include phylogenetically important clades as well as clades with current or forthcoming genomic resources.

In vascular plants, there is a striking disparity in chromosome number between homosporous and heterosporous lineages (Klekowski and Baker, 1966): homosporous lineages have high chromosome numbers and often larger genomes, compared to heterosporous ones (Leitch and Leitch, 2013; Figure 1). Thus, several studies have asked why homosporous plants have so many chromosomes (Klekowski and Baker, 1966; Klekowski, 1969; Nakazato et al., 2008; Barker and Wolf, 2010). However, homospory is the ancestral condition from which heterospory has been derived several times (Bateman and DiMichele, 1994; Figure 2). Given that the character state of high chromosome numbers in homosporous plants is ancestral (Clark et al., 2016; Carta et al., 2020), it makes more sense evolutionarily to ask why heterosporous plants have comparably so few chromosomes.

This review covers the life history differences between heterosporous and homosporous vascular plants, previous work on chromosome number and genome size in each lineage, and known mechanisms of genomic and chromosomal change. We address the current evidence for the processes controlling genome evolution and the variation in genome downsizing rates between homosporous and heterosporous plants, as well as how the pattern of spore production might be related to these mechanisms. We conclude with prospects for research on this relationship and what types of data will be needed to solve a mystery that has haunted botanists for over half a century.

## Life History of Homosporous and Heterosporous Vascular Plants

All seed plants, and some spore-dispersed plants, are heterosporous (Figure 2). They produce two different types of sporangia, resulting in the small microspores (sperm-producing) that develop to become microgametophytes and larger megaspores (egg-producing) that develop to form the megagametophytes. In seed plant microsporogenesis, all four meiotic products are retained and grow into pollen grains. In contrast, during megasporogenesis, only one of the four meiotic products survives. There are some exceptions to this process, including apomictic species where a polar body fertilizes the megaspore, as well as species where the polar bodies develop into endosperm (e.g., Schmerler and Wessel, 2011; Noyes and Givens, 2013).

Homosporous plants comprise most ferns, some lycophytes, as well as bryophytes (the latter are non-vascular). They produce a single type of spore that germinates into a gametophyte theoretically capable of producing both egg and sperm. Most ferns are homosporous, but one clade of aquatic ferns (Salviniales) has evolved to become heterosporous. Heterospory also evolved independently in the lycophytes. It is estimated that heterospory has evolved at least 11 times throughout the history of land plants (Bateman and DiMichele, 1994). However, we only have three independent lineages of extant heterosporous plants (Figure 2) with which to test patterns of associated characters. These extant heterosporous lineages provide us with natural replicates to study the evolution of this trait, as all three lineages have homosporous sister lineages for comparison.

## History of Chromosome Research in Plants

The study of plant chromosomes dates back to the nineteenth century when researchers began using microscopy to study cell biology. In the 1880s, the stages (Flemming, 1965) and timing (Strasburger, 1894) of cell division were first described, although the exact nature and importance of chromatin were not yet understood (Volkmann et al., 2012). In 1888, von Waldeyer-Hartz coined the term chromosome, and the structure and heritability of chromosomes became established soon after (Strasburger, 1894; Cremer and Cremer, 1988; Winkelmann, 2007). Around the turn of the twentieth century, researchers noticed plants with doubled numbers of chromosomes (e.g., *Oenothera*, Lutz, 1907; Strasburger, 1910). This work resulted in the theory that genome doubling restored fertility to hybrid plants (Winge, 1917). During the following decades, additional work extended the knowledge base of plant cytology, with a wide range of heterosporous study systems including *Nicotiana* (Clausen and Goodspeed, 1925), *Oenothera* (Gates et al., 1929), *Viola* (Clausen, 1927), *Gossypium* (Skovsted, 1935), and the Salicaceae (Blackburn and Harrison, 1924).

Around the same time, work also began on homosporous fern genetics (Lang, 1923; Andersson-Kottö, 1927, 1929, 1938). These initial studies provided the first chromosome counts and crossing experiments in ferns; importantly, they presented the theory that ferns with high chromosome numbers had diploid, not polyploid, inheritance (Andersson-Kottö, 1929, 1938; Haufler, 2002). In the 1950 book, *Problems of cytology and evolution in the Pteridophyta* (Manton, 1950), the chromosome numbers of about 100 species of ferns were published for the first time. This work provided an important reference for fern cytology and helped establish the importance of base chromosome numbers in classification (Manton and Sledge, 1954).

In 1966, the first connection was made between the differences in chromosome numbers of homosporous and heterosporous ferns. Klekowski and Baker (1966) used previously published chromosome counts to show that within ferns and lycophytes, homosporous species had an average sporophytic count of 2*n* = 115, whereas for heterosporous species it was 2*n* = 27.24. In comparision, in angiosperms (all heterosporous) was 2*n* = 31.98. These findings have been substantiated by modern methodologies. Additionally, researchers have been able to evaluate the chromosome number and genome size for the ancestors of angiosperms and spore-dispersed plants. The ancestral gametophytic chromosome number for angiosperms has been estimated to be *n* = 7 (Carta et al., 2020), whereas in homosporous ferns this is estimated to have been *n* = 22 (Clark et al., 2016). These findings indicate that chromosome number may be influenced by the shift in life history to heterospory, although the mechanisms are still unclear (Clark et al., 2016; Carta et al., 2020; Fujiwara et al., 2021; Szövényi et al., 2021).

We downloaded (in January 2021) chromosome counts from 377,715 records in the Chromosome Counts Database version 1.47 (Rice et al., 2015). In addition, we examined data on genome size from the Plant DNA *C*-values Database (Pellicer and Leitch, 2020). Here, we also analyze the data, removing likely recent polyploid species from the analysis to include only the base chromosome number for each species. We did this by allowing for aneuploid and dysploid change, and retaining species within a genus, with up to 1.2 times the minimum chromosome number recorded for the genus. We show chromosome numbers (Figures 1A,B) and genome sizes (Figures 1C,D) for homosporous and heterosporous vascular land plants. We present box plots for four heterosporous and two homosporous lineages (Figures 1A,C) and then also compare the distributions of all homosporous plants versus all heterosporous plants (Figures 1B,D). Note that our plots (Figures 1B,D) include histograms and kernel density because the sample sizes were very different between the groups and binning sizes made comparisons difficult.

On average, homosporous plants have more than three times as many chromosomes and more than three times the genome size as heterosporous plants (Figure 1). In all four heterosporous lineages (heterosporous lycophytes and ferns, gymnosperms, and angiosperms; Figure 2), there is a reduction in chromosome number. The pattern for genome size, however, is slightly more complex. Gymnosperms are anomalous with large genomes, despite relatively low chromosome numbers. It has been hypothesized that at least some gymnosperms have an unusually high density of long terminal repeat retrotransposons (LTR-RTs), responsible for their large genome sizes (Nystedt et al., 2013). Considering both chromosome number and genome size, there are differences in genome architecture and evolution between homosporous and heterosporous plants. What remains to be discovered are the genetic mechanisms that control these differences, and how they are influenced by life history.

## How Can the Pattern of Spore Production Be Related to Chromosome Number?

At first glance, it seems unlikely that aspects of chromosome number could be related to the spore types. Why would an evolutionary transition to heterospory accompany the derived character state of small genomes and low chromosome numbers? This question has challenged botanists for decades, and no simple hypothesis has yielded a satisfactory explanation. In the 1960s, starting with the observation of high chromosome numbers in homosporous pteridophytes (Klekowski and Baker, 1966), Klekowski (1969, 1972, 1973) developed a testable hypothesis for a causal relationship, based on the different reproductive modes that can occur in heterosporous versus homosporous plants. In heterosporous plants, reproduction can proceed *via* sporophytic selfing (sperm and egg from the same parent plant, but different gametophytes) or outcrossing (sperm and egg from different plants). In homosporous plants, both sporophytic selfing and outcrossing are possible, but an additional reproductive mode can occur called gametophytic selfing (Haufler et al., 2016). This extreme form of self-fertilization occurs when an egg is fertilized by a sperm from the same gametophyte. A gametophyte generates gametes *via* mitosis, so all gametes are genetically identical; therefore, a sporophyte created from gametophytic selfing will be homozygous at every locus in the genome.

Klekowski proposed that ferns primarily reproduced *via* gametophytic selfing and consequently would have high genetic load if they were diploid. Therefore, ferns with high chromosome numbers must have polyploid, not diploid, inheritance (Klekowski and Baker, 1966), directly opposing early work on fern genetics (Andersson-Kottö, 1929). Klekowski proposed that if these homosporous species became polyploid through hybridization (allopolyploidy) then diverse alleles could be maintained across the different parental genomes, reducing genetic load despite extreme inbreeding (Klekowski, 1976). This occurs *via* homoeologous heterozygosity: when matching chromosomes (homeologs) from different parental genomes in a hybrid carry distinct alleles (Glover et al., 2016). Furthermore, if the homoeologous chromosomes could pair, even a sporophyte that is homozygous at all homologous loci could create genetically variable meiotic products (Klekowski, 1972, 1973). Thus, the increased chromosome sets were needed to overcome the extreme mating system in homosporous plants.

Throughout the 1970s, researchers gathered data that seemed to support Klekowski’s hypothesis. Evidence from chromosome studies, breeding studies, and genetic analyses seemed to indicate that homoeologous recombination was possible in homosporous ferns (Hickok and Klekowski, 1973; Klekowski and Hickok, 1974; Chapman et al., 1979; Hickok, 1979). Most of this work came from lab experiments, but starting in the 1980s, researchers began to examine how homosporous plants behaved in natural settings. The first observation was that homosporous ferns appeared to be mostly outcrossing (Haufler and Soltis, 1984; Gastony and Gottlieb, 1985; Wolf et al., 1987), evidence that did not support high levels of gametophytic selfing which would be required to provide the selective pressures under Klekowski’s hypothesis. Furthermore, studies applying electrophoresis of enzymes showed that even ferns with high chromosome numbers expressed the typical diploid number isozymes (Wolf et al., 1987). This was subsequently confirmed with genomic sequencing in the homosporous fern *Ceratopteris* (Marchant et al., 2019). Such evidence suggested that even species that appear to be polyploid behave genetically as diploids (Haufler and Soltis, 1986; Haufler, 1987, 1989) and that gametophytic selfing might actually be rare in homosporous ferns (Haufler et al., 2016).

Missing from this research, however, was a robust evolutionary framework for comparative analyses; such a phylogenetic perspective is critical for inferring the evolutionary processes that influence genome structure. Reconstructing the phylogeny for land plants started in the 1990s when *rbcL* was developed as a phylogenetic marker for angiosperms, followed shortly by large-scale phylogenetic analyses of ferns (Pryer et al., 1995). By early in the twenty-first century, there was a good working hypothesis for relationships among the major groups of ferns (Pryer et al., 2004). Researchers also began assembling plant genomic resources. Sequencing the *Arabidopsis* genome (The Arabidopsis Genome Initiative, 2000) was an important first step, soon followed by many more seed plant genomes. The 1000 Plant Transcriptomes project was a major step in generating genetic data for species across the land plant phylogeny (One Thousand Plant Transcriptomes Initiative, 2019). The first linkage map for a homosporous fern was published for *Ceratopteris richardii* (Nakazato et al., 2006). However, the first fern genomes were not completed until 2018 for heterosporous ferns (Li et al., 2018), and 2019 for homosporous *Ceratopteris* (Marchant et al., 2019). Whole genome sequences are also published for liverworts (Bowman et al., 2017), mosses (Rensing et al., 2008), and hornworts (Li F. W. et al., 2020). These bryophyte genomes provide us with an outgroup for all vascular land plants. The current phylogenetic, genomic, and transcriptomic resources are very close to providing the resources necessary to answer some of the old questions regarding heterosporous and homosporous genomes and chromosome evolution (Fujiwara et al., 2021; Szövényi et al., 2021).

## Mechanisms of Genomic and Chromosomal Change

Despite limited phylogenetic, genomic, and transcriptomic resources, there is a growing body of research on broad patterns of chromosomal and genomic evolution between homosporous and heterosporous plant genomes. In most groups of organisms, there is not a good correlation of chromosome number with genome size. The reasons for this are complex and involve the, often rapid, loss of genetic material after a whole-genome duplication (WGD) event (Leitch and Bennett, 2004). In contrast, some studies suggest that genome size is positively correlated with chromosome number in homosporous ferns (Nakazato et al., 2008; Bainard et al., 2011; Clark et al., 2016; Fujiwara et al., 2021), indicating that ferns have fundamentally different mechanisms of genome downsizing compared to other organisms (Barker and Wolf, 2010; Leitch and Leitch, 2012). Here, we cover some hypotheses about the differences in genome downsizing, architecture, and chromosome structure between homosporous and heterosporous plants.

### Genome Downsizing

In angiosperms, polyploidy (and associated WGD) has played a major role in the evolution of the vast majority of species, including both recent and ancient WGD events (Cui et al., 2006; Van de Peer et al., 2009, 2017). However, the long-term evolutionary effects of WGD are complex, and polyploid lineages include a mix of evolutionary dead ends as well as critical lineages that survive, exploit new niches, and radiate, perhaps as a consequence of polyploidy (Van de Peer et al., 2017). When polyploids are initially formed they have twice as many genes as they typically need, relaxing selection pressure on retention of the duplicated copies. This, combined with a documented breakdown in the meiotic process in polyploids (Ramsey and Schemske, 2002; Chester et al., 2012) can lead to rapid loss of chromosomal segments, resulting in downsizing of the genome (Leitch and Bennett, 2004; Li Z. et al., 2020; Bowers and Paterson, 2021). Studies of recent allotetraploids reveal extensive chromosomal variation, including intergenomic translocations, which all appear to be part of the process of rapid genome downsizing in angiosperms (Lim et al., 2008; Chester et al., 2012). These genomic changes ultimately lead to the restoration of disomic inheritance and bivalent chromosome pairing in lineages that have experienced WGD (Li Z. et al., 2020).

Much less is known about genomic change following WGD in homosporous plants, which has yet to be studied extensively (Szövényi et al., 2021). Overall, it is thought that genome downsizing proceeds slower in ferns than angiosperms (Barker and Wolf, 2010), and diploidization in ferns may be driven by pseudogenization and/or gene silencing rather than gene loss (Haufler, 1987; Barker, 2013; Clark et al., 2016). The latter process potentially leads to larger genomes, or rather genomes that do not decrease in size following polyploidization. Differences in downsizing rates may be a consequence of several processes. If fern genome downsizing is slower, it could be because ferns lose fewer chromosomal regions per generation. However, the cause could also be a reduction in the size, not the rate, of the chromosomal segments being lost. Recent work suggests that the rate of genome evolution may influence speciation rate. This is supported by high rates of genome evolution in the species-rich Polypodiales, which contain over three-quarters of extant fern diversity (PPG, 2016; Fujiwara et al., 2021). The mechanisms influencing fern genome evolution, however, are less clear. This illustrates the need for experiments that track chromosomal changes following polyploidy in ferns, and compare this to genome downsizing in heterosporous plants (Lim et al., 2008; Chester et al., 2012). Understanding the processes that influence genome architecture and the rate of genomic change between lineages may help advance our knowledge of broad-scale speciation dynamics of all land plants (Fujiwara et al., 2021).

### Genome and Chromosome Architecture

In addition to genome size, heterosporous and homosporous genomes have some critical differences in structure and composition. Our current knowledge of these differences in homosporous plants is limited to genome skimming and transcriptome studies, but these are important to inform future comparative genomic work. In heterosporous seed plants (angiosperms and gymnosperms), one aspect of genome size variation is transposable elements, such as LTR-RTs, which increase in copy number over time and can cause even diploid genomes to become very large (Wendel et al., 2016). Baniaga and Barker (2019) found that LTR-RT are also present in homosporous fern genomes, but have much older insertion times than in angiosperm taxa; this means that the LTR-RTs of homosporous ferns have had time to increase in copy number, inflating genome size. In addition, they found that heterosporous ferns (*Azolla* and *Salvinia*) and lycophytes (*Selaginella*) have LTR-RTs insertion times more similar to angiosperms than to homosporous ferns (Baniaga and Barker, 2019). These findings are consistent with the observation that homosporous fern genomes have a greater proportion of repeat elements than angiosperm genomes (Wolf et al., 2015). Baniaga and Barker (2019) hypothesized that LTR-RTs are associated with high methylation in homosporous ferns (Takuno et al., 2016): LTR-RTs are silenced by methylation, and methylation can also silence genes on the same chromosome because it targets repeat elements. Homosporous ferns may ultimately not purge these methylated and silenced LTR-RTs and other genes, leading to their large genome size (Baniaga and Barker, 2019). However, long-read sequencing and high-quality genome assemblies are needed to understand the evolutionary dynamics of repeat elements in homosporous ferns. Future comparative genomic work could investigate the role of mating systems and life history on repeat elements in homosporous and heterosporous genomes (Baniaga and Barker, 2019). Additionally, genomic data from a homosporous lycophyte (Lycopodiales) is needed to compare gene structure across homosporous lineages.

The distribution of genes and repeat elements along chromosomes also differs between heterosporous and homosporous plants. The structure of angiosperm chromosomes seems to be fairly well conserved, with most genes occurring between the repeat-rich pericentromeric and telomeric regions (Szövényi et al., 2021). Comparatively, in seed-free plants, genes and repeats are interspersed along the chromosomes rather than separated into repeat-rich and gene-rich areas (Banks et al., 2011; Liang et al., 2020). However, this has only been studied in mosses and lycophytes (Banks et al., 2011; Shakirov and Shippen, 2012; Bowman et al., 2017; Li F. W. et al., 2020). Chromosome-scale genome assemblies are needed to investigate such structure in homosporous ferns, as well as understand the variation in chromosome structure between homosporous lineages.

Variation in chromosome size is another major difference between heterosporous and homosporous plants, with a 3,100-fold variation in angiosperms compared to 31-fold in homosporous ferns and lycophytes (Clark et al., 2016). Our understanding of the mechanisms controlling chromosome size, particularly in ferns, is limited (Szövényi et al., 2021). Schubert and Oud (1997) showed that there is an upper limit to chromosome arm length in angiosperms, and Liu et al. (2019) found support for conservation of chromosome size within the fern genus *Asplenium*. Work in angiosperms has found that mitotic divisions may fail when the chromosome arm: spindle length ratio is above a certain point (Schubert and Oud, 1997). Investigating this ratio in homosporous and heterosporous ferns and lycophytes could be a fruitful avenue of study, as there may be fundamental differences in cell division between these lineages and seed plants. Overall, investigating patterns of both chromosome- and genome-scale structure will greatly benefit our understanding of both heterosporous and homosporous genomes.

## Discussion: Comparative Analyses of Homospory and Heterospory

If we are to understand the relationship between genome architecture and spore type, then the necessary studies must begin with comparative analyses from a phylogenetic perspective. Given the three independent origins of heterospory (Figure 2), we ask what genomic characteristics are uniquely shared on the three branches subtending these heterosporous clades? Understanding *what* parts of the genome have changed following a transition to heterospory is critical for developing hypotheses explaining *why* these evolutionary steps have occurred. Within a few years, we should have sufficient numbers of homosporous genomes to search for statistically significant differences in gene family expansion and contraction, signatures of selection, trends in rates of pseudogenization, distribution and insertion rates of various groups of transposable elements and retrotransposons, and comparative patterns of synteny and general genome architecture. This work would then inform novel hypothesis-driven approaches that could include comparative analyses of genes related to meiosis and chromosome structure, such as those associated with spindle fiber genes, cell cycle genes, telomere structure, centromere structure, kinetochores, and recombination. We would also benefit from a return to chromosome analysis, but with genomic perspectives, using approaches such as fluorescent *in situ* hybridization combined with chromosome painting (e.g., Schubert and Lysak, 2011; Šimoníková et al., 2019).

We currently have the resources to explore some potential drivers of genomic change in homosporous ferns. To explain the observed chromosomal differences between homosporous and heterosporous genomes, a few theories have been proposed based on the disparate life histories of these two groups. For example, even if gametophytic selfing is not particularly common in homosporous ferns, it could still play a role in genome evolution, especially in chromosome structure. Marchant (2019) suggested that if there is a major chromosomal change in meiosis, the resulting gametophyte could successfully self-fertilize because both sperm and egg would carry the same mutation. All chromosomes would pair without issue, fixing a new chromosome arrangement. Such an event could not occur in a heterosporous plant with obligate gametophytic outcrossing (Marchant, 2019). Gametophytic selfing would reduce negative selection on chromosomal malformations or other components such as repetitive elements (Baniaga and Barker, 2019) in homosporous plants, potentially leading to larger and more dynamic genomes than in heterosporous plants. Similarly, dysploidy and polyploidy might be more successful in lineages with gametophytic selfing, such as Ophioglossaceae, which have underground gametophytes (Soltis and Soltis, 1986; Hauk and Haufler, 1999), as well as very large genomes (Bainard et al., 2011).

This hypothesis proposed by Marchant (2019) suggests that homosporous fern genomes are more stable following WGD, due to gametophytic selfing. This would relax evolutionary pressures to downsize the genome, as all chromosomes could pair without issue, resulting in more consistent successful gamete production. One could test this hypothesis by examining natural and artificial polyploids to see if genomic segments are lost as fast as they are in seed plants (e.g., Lim et al., 2008; Chester et al., 2012). Furthermore, chromosome structural analyses will be needed to test Marchant’s (2019) hypothesis for fixation of chromosomal changes *via* gametophytic selfing. This could be accomplished through comparative karyotype analysis or computational analysis of synteny in a series of sister taxa.

Another potential driver of chromosome evolution in plants is transmission ratio distortion (TRD): the preferential inheritance of one allele over the other (Huang et al., 2013). TRD can be caused by several mechanisms, including germline selection (Hastings, 1991) and meiotic drive (Pardo-Manuel et al., 2001). Both of these processes can influence genome structure by preferentially selecting for a certain gene or chromosome structure, although they affect homosporous and heterosporous plants differently. During megasporogenesis in most heterosporous angiosperms, four cells result from meiosis but only one of the two outer cells survives to become the megagametophyte; the remaining polar bodies die. Certain genes, chromosomes, or portions of chromosomes can be preferentially transported to these outer cells, increasing the probability of being passed to the next generation. There are physical attributes of chromosomes that help to transport all or a portion of a chromosome to the outer cells during meiosis (Burt and Trivers, 2009). For example, there is a bias against inversions and for deletions, because smaller chromosomes move faster along the spindle and therefore are more likely to end up in the outer two cells at the end of meiosis (Burt and Trivers, 2009).

In most homosporous ferns, spores are produced from one archesporial cell, which goes through four rounds of mitosis to produce 16 cells; then, these mother cells go through one round of meiosis to produce 64 viable spores (Manton, 1950). Because all meiotic products survive, meiotic drive (as it exists in angiosperms) does not occur in homosporous plants. Therefore, there is no selection pressure on chromosome size or composition during gamete formation, although it may occur at other points such as germline mitosis (Hastings, 1991; Clark et al., 2016). The effect of TRD on genome composition in heterosporous plants could be part of the reduction of chromosome number and genome size in angiosperms. Comparative analyses are needed to measure the extent and affect of TRD on genome composition in homosporous and heterosporous plants.

The presence of TRD in homosporous ferns could be tested using reduced representation sequencing such as restriction site-associated DNA sequencing (RADseq). Two parental species could be crossed to form a hybrid, then spores from the hybrid germinated. Using RADseq, the parents, hybrid, and gametophytes derived from the hybrid would be genotyped. Because ferns have an independent gametophyte stage, meiotic products can be assessed directly without the need for a test cross. Thus, progeny arrays of gametophytes could be genotyped to estimate meiotic product ratios and determine if certain alleles are being preferentially transmitted.

Finally, there are several natural study systems to leverage for work on genomic change following a transition to heterospory. As mentioned previously, there are three independent evolutions of heterospory in land plants: heterosporous ferns (Salviniales), lycophytes (Selaginellales and Isoetales), and the seed plants. The heterosporous ferns and lycophytes both have sister homosporous lineages that would be an ideal comparative system. By first using these two groups to understand how heterospory influence genome evolution in spore-dispersed plants, we could build a foundation to compare homosporous fern genomes to heterosporous seed plants genomes. Another natural system for exploring the evolution of heterospory is *Pteris platyzomopsis* (Pteridaceae). This fern appears to be an example of incipient heterospory; it produces spores in two size classes and has dioecious gametophytes, although egg-producing gametophytes will also produce antheridia after a certain period of time (Tryon, 1964). *Pteris platyzomopsis* has a base chromosome number of *n* = 38 (Tryon and Vida, 1967), which is similar to other homosporous species, but nothing is known about its genome size or structure. More research is also needed on the life history of this species. Incorporating *P. platyzomopsis* in comparative work will be important to understand the evolution of heterospory at the genomic level.

## Conclusion

The disparity between chromosome number in homosporous and heterosporous plants is one that has challenged scientists for decades (e.g., Klekowski and Baker, 1966). To conduct the necessary comparative analyses, more high-quality genomes from homosporous ferns and lycophytes are needed, which is something many authors have been seeking for almost 20 years (Pryer et al., 2002; Sessa et al., 2014; Wolf et al., 2015; Kuo and Li, 2019). Today, however, our genomic resources are growing rapidly and in a few years, there may be enough homosporous genomes to begin comparative analyses with heterosporous lineages (Kuo and Li, 2019; Szövényi et al., 2021). When investigating broad-scale patterns in vascular plant evolution, it is important to include an evolutionary perspective. In this review, we examined the multiple origins of heterospory in plants and considered which traits might be affecting their chromosome numbers, genome size, and genome composition. A combination of new data and new ways of looking at the problem may determine which factors are involved and which assumptions we have overlooked.

## Author Contributions

PW and SK conceived of the project. CR performed the data analyses and figure design. All authors participated in writing and editing the manuscript.

## Conflict of Interest

The authors declare that the research was conducted in the absence of any commercial or financial relationships that could be construed as a potential conflict of interest.

## Publisher’s Note

All claims expressed in this article are solely those of the authors and do not necessarily represent those of their affiliated organizations, or those of the publisher, the editors and the reviewers. Any product that may be evaluated in this article, or claim that may be made by its manufacturer, is not guaranteed or endorsed by the publisher.
